# Systematic benchmarking of computational methods to identify spatially variable genes

**DOI:** 10.1186/s13059-025-03731-2

**Published:** 2025-09-18

**Authors:** Zhijian Li, Zain M.Patel, Dongyuan Song, Sai Nirmayi Yasa, Robrecht Cannoodt, Guanao Yan, Jingyi Jessica Li, Luca Pinello

**Affiliations:** 1 Gene Regulatory Observatory, Broad Institute of MIT and Harvard, Cambridge, MA USA; 2 Molecular Pathology Unit, Krantz Family Center for Cancer Research, Massachusetts General Hospital, Boston, MA USA; 3 Department of Genetics and Genome Sciences, University of Connecticut Health Center, Farmington, CT USA; 4 Data Intuitive, Lebbeke, Belgium; 5 Data Mining and Modelling for Biomedicine Group, VIB Center for Inflammation Research, Ghent, Belgium; 6 Department of Applied Mathematics, Computer Science, and Statistics, Ghent University, Ghent, Belgium; 7 Department of Statistics and Data Science, University of California, Los Angeles, CA USA; 8 Department of Computational Mathematics, Science and Engineering, Michigan State University, East Lansing, MI USA; 9 Biostatistics Program, Public Health Sciences Division, Fred Hutchinson Cancer Center, Seattle, WA USA

**Keywords:** Benchmarking, Spatially variable genes, Simulation, Spatial omics, Visium, MERFISH

## Abstract

**Background:**

Spatially resolved transcriptomics offers unprecedented insight by enabling the profiling of gene expression within the intact spatial context of cells, effectively adding a new and essential dimension to data interpretation. To efficiently detect spatial structure of interest, an essential step in analyzing such data involves identifying spatially variable genes (SVGs). Despite researchers having developed several computational methods to accomplish this task, the lack of a comprehensive benchmark evaluating their performance remains a considerable gap in the field.

**Results:**

Here, we systematically evaluate 14 methods using 96 spatial datasets and 6 metrics. We compare the methods regarding gene ranking and classification based on real spatial variation, statistical calibration, and computation scalability and investigate the impact of identified SVGs on downstream applications such as spatial domain detection. Finally, we explore the applicability of the methods to spatial ATAC-seq data to examine their effectiveness in identifying spatially variable peaks (SVPs). Overall, SPARK-X outperforms other benchmarked methods and Moran’s I achieves a competitive performance, representing a strong baseline for future method development. Moreover, our results reveal that most methods are poorly calibrated, and more specialized algorithms are needed to identify spatially variable peaks.

**Conclusions:**

Our benchmarking provides a detailed comparison of SVG detection methods and serves as a reference for both users and method developers.

**Supplementary Information:**

The online version contains supplementary material available at 10.1186/s13059-025-03731-2.

## Background

Recent years have witnessed significant progress in spatially resolved transcriptome profiling techniques that simultaneously characterize cellular gene expression and their physical position, generating spatial transcriptomic (ST) data. The application of these techniques has dramatically advanced our understanding of disease and developmental biology, for example, tumor-microenvironment interactions [1], tissue remodeling following myocardial infarction [2], and mouse organogenesis [3], among others.

**Fig. 1:**
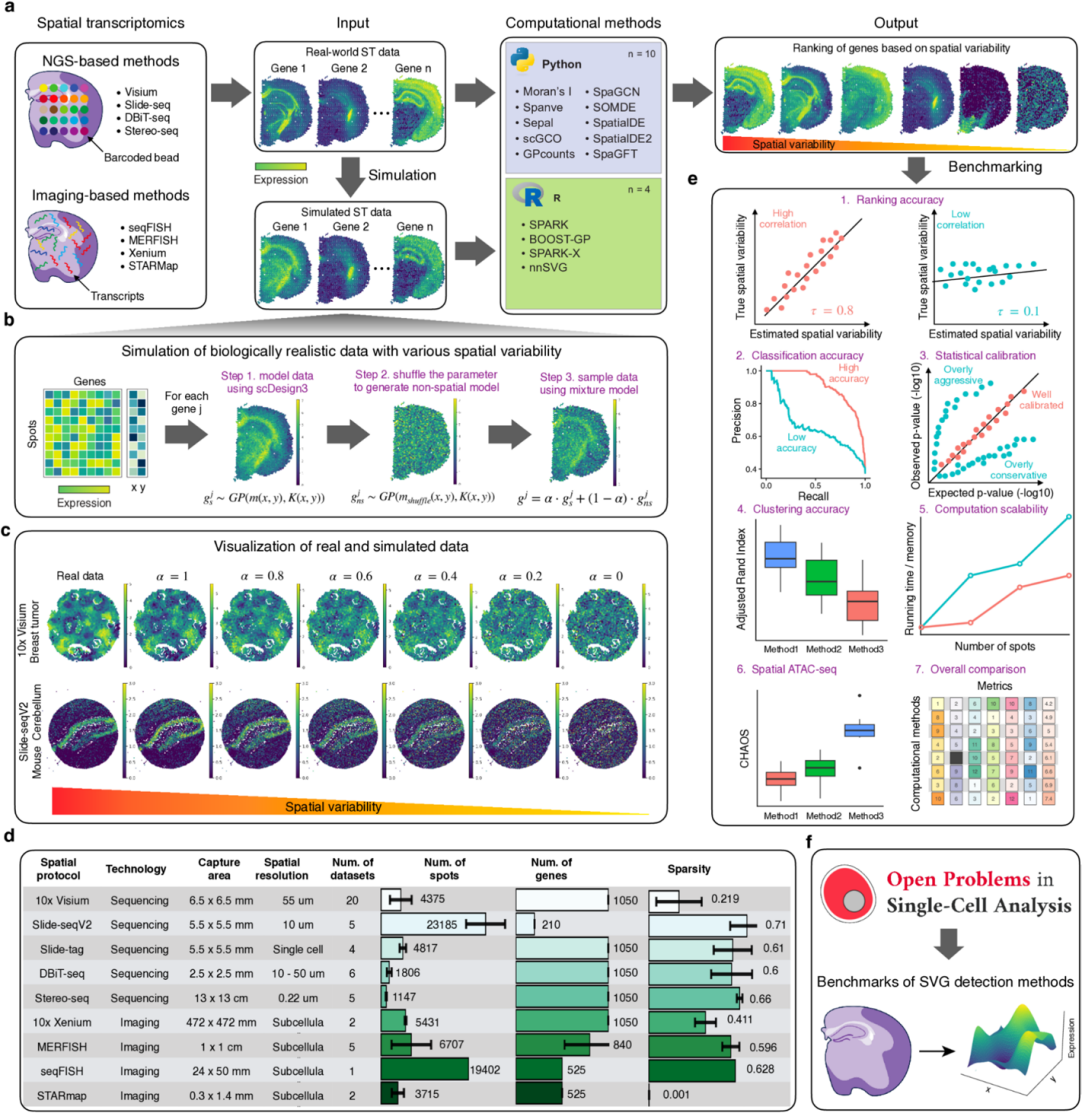
Overview of spatially variable gene identification, data simulation, evaluation metrics, and statistics of datasets. **a** Left: schematic showing different protocols for profiling spatially resolved transcriptomics. Middle: visualization of gene expression with various expression patterns in spatial space using real-world and simulated data. Right: computational methods to rank genes based on measured spatial variability. **b** Workflow of using scDesign3 and real-world data to generate realistic spatial transcriptomics data with controlled spatial variability. **c**Visualization of real-world and simulated data using 10 × Visium or Slide-seq V2 as reference. The first column shows real expression, and the other columns show simulated expression ordered by spatial variability. **d** Summarization of the 50 real-world datasets used to simulate data. Bar plots show the number of spots, the number of genes, and the sparsity for each dataset. **e** Methods are evaluated by different metrics, including ranking accuracy, classification accuracy, statistical calibration, clustering accuracy, computational scalability, effectiveness for spatial ATAC-seq, and overall performance. **f** Benchmarks of SVG detection methods at the Open Problems in Single-Cell Analysis platform

Spatial transcriptome profiling methods are broadly categorized into two groups, i.e., sequencing-based (including 10 × Visium [4]; Slide-seq [5, 6]; HDST [7]; STARmap [8]) and imaging-based (including seqFISH [9] and MERFISH [10]) (Fig. 1a). They vary in terms of the number of genes and spatial resolution. Specifically, sequencing-based assays usually provide genome-wide gene expression measurements through spots profiling multiple cells, thus precluding the possibility of delineating expression at the single-cell level. At the same time, the imaging-based methods can generate sub-cellular resolution data but can only detect a subset of genes (30–300). Due to these differences in the number of genes and spatial resolution, distinct computational methods and algorithms are required for the downstream analysis of each data type. In the case of sequencing-based profiles, an important task involves associating cell types with spatial locations through cell-type deconvolution, often leveraging paired single-cell RNA-seq data to compensate for the low spatial resolution [11–13]. On the other hand, for imaging-based profiles, the initial step involves performing cell segmentation to accurately delineate the boundaries of individual cells [14].

One common task for all ST profiles, regardless of the employed protocols, is to identify genes that show significant associations between the spatial distribution of the cells and their expression levels [15] (Fig. 1a). These genes are defined as spatially variable genes (SVGs). Identifying SVGs from ST data has helped researchers better understand developmental gradients, cell signaling pathways, and tumor microenvironments. Additionally, SVGs have become critical to downstream tasks such as identifying and detecting spatial domains within tumors and tissues [16]. To detect SVGs, researchers have developed various computational methods by incorporating the spatial context into the analysis [17–22]. As the number of methods keeps increasing, it becomes challenging for users to select the most effective approach for identifying SVGs from their respective ST profiling technique (e.g., 10 × Visium, MERFISH). Previous benchmarking studies have typically compared no more than seven computational methods [23–25], significantly fewer than the currently available approaches (*n* > 14) and not covering comprehensively all the available spatial profiling technologies. Furthermore, since obtaining ground truth from real-world ST profiles is not feasible, these studies have relied on simulation data to evaluate the accuracy of each method in detecting SVGs. However, the simulation data were generated either using predefined spatial clusters [26, 27] or with a limited number of expression spatial patterns, e.g., *spots* where the expression forms round contours and *linear* where the expression forms rectangular shapes [24]. Consequently, the simulated data generated in these studies do not fully capture observed biological patterns nor account for the variability across tissue types and spatial technologies. Therefore, these approaches led to inflated performance metrics compared to those that might be obtained in more realistic settings. Taken together, there is a clear need for comprehensive benchmarking that incorporates more methods and employs enhanced and realistic simulation strategies to capture biologically plausible patterns of interest. For these reasons, we want to provide here a more robust and unbiased evaluation of the available methods for detecting SVGs in ST profiles, enabling researchers to make informed decisions when selecting the most appropriate computational methods for their analyses.

In this work, we benchmarked 14 computational methods for identifying SVGs (see Table 1). To evaluate these methods, we simulated a number of realistic datasets with diverse patterns derived from real-world ST data using scDesign3 [28], a state of the art simulation framework for single cell assays. We compared the methods regarding gene ranking and classification based on real spatial variation, statistical calibration, and computation scalability. We also investigated the impact of identified SVGs on downstream applications such as spatial domain detection. Finally, we explored the applicability of the methods to spatial ATAC-seq data to examine their effectiveness in identifying spatially variable peaks (SVPs). Our benchmarking results revealed that, on average, *SPARK-X* was the best-performing method across our six metrics. Interestingly, we observed that all methods except for *SPARK* and *SPARK-X* produced inflated *p*-values, indicating that they are statistically poorly calibrated. Regarding scalability, *SOMDE* performed the best across memory usage and running time. Additionally, we found that using SVGs generally improved spatial domain detection compared to highly variable genes. Moreover, all methods but *SpatialDE2* performed poorly in detecting spatial variable peaks (SVPs) for spatial-ATAC-seq, indicating the need for more specialized methods for this task. The results are also available on the Open Problems, a living and extensible benchmarking platform (https://openproblems.bio/results/spatially_variable_genes). Overall, our benchmarking provides a detailed comparison of SVG detection methods and serves as a reference for both users and method developers.

## Results

### Overview of computational methods for detection of spatially variable genes

The identification of spatially variable genes (SVGs) in spatial transcriptomics data requires computational methods that integrate both gene expression levels and spatial information (cellular or subcellular level), unlike the detection of highly variable genes (HVGs) in traditional single-cell RNA sequencing data. A common and straightforward approach is to build a k-nearest-neighbor (KNN) graph where each node represents a spatial spot, and the edges between nodes represent the spatial proximity of spots. SVGs are identified by combining this spatial neighbor graph with gene expression profiles. For instance, *Moran’s I*, a classic spatial autocorrelation metric, estimates the correlation coefficient of the expression between a spot and its neighbors [29, 30]. Similarly, *Spanve* quantifies the divergence in gene expression distributions between randomly and spatially sampled locations using Kullback–Leibler (KL) divergence [31]. A higher correlation or distribution divergence indicates that the gene is more likely to have a non-random spatial pattern. *scGCO* utilizes a hidden Markov random field (HMRF) to capture the spatial dependence of each gene’s expression levels and uses a graph cut algorithm to identify the SVGs [21]. *SpaGCN* first builds a graph by integrating gene expression, spatial location, and histology information (when available) and then clusters the spots using a graph convolutional network (GCN) [32]. SVGs are identified by differential expression (DE) analysis on the obtained clusters [22]. *SpaGFT* constructs a KNN graph of spots based on their spatial proximity and then transforms each gene’s expression to the frequency domain; genes with low-frequency signals tend to have less random spatial patterns [33]. *Sepal* uses a diffusion model to assess the degree of randomness of each gene’s spatial expression pattern and ranks the genes accordingly [34].

Another strategy to incorporate spatial information involves utilizing a kernel function that takes spatial distance as input and computes a covariance matrix to capture the spatial dependency of gene expression across locations. This covariance matrix represents a prior of the underlying spatial pattern. One of the pioneer methods is *SpatialDE* [18], which models the normalized expression data using non-parametric Gaussian Process (GP) regression and tests the significance of the spatial covariance matrix for each gene by comparing the fitted models with and without the spatial covariance matrix. *SpatialDE2* [35] further extends this framework by providing technical innovations and computational speedups. *SPARK* [19] proposes another extension by modeling the raw counts with a generalized linear model based on the over-dispersed Poisson distribution. It provides a more robust statistical approach (based on the Cauchy combination rule [36]) to assess the significance of the identified SVGs. In contrast, *BOOST-GP* uses a zero-inflated negative binomial (ZINB) distribution to model the read counts and infers the model parameters via a Markov Chain Monte Carlo (MCMC) algorithm [37]. Similarly, *GPcounts* [38] models the counts with a negative binomial (NB) distribution and estimates the model parameters using variational Bayesian inference to improve computational efficiency. Notably, *SPARK-X* stands as an exception by directly comparing the expression covariance matrix and the spatial distance covariance matrix, yielding substantial computational efficiency gains [20].

In addition, two hybrid methods, namely *nnSVG* and *SOMDE*, have been developed to integrate graph and kernel approaches to capture the spatial dependence between spatial spots. The *nnSVG* method utilizes a hierarchical nearest-neighbor GP to model the large-scale spatial data [39], providing computational efficiency gains over the standard GP used in SpatialDE. On the other hand, *SOMDE* employs a self-organizing map (SOM) to cluster neighboring cells into nodes and subsequently fits node-level spatial gene expression using the GP model to identify SVGs [40]. Both methods reduce the computational complexity of kernel approaches by leveraging a spatial graph, which significantly improves their scalability. We summarized the key features of the 14 methods in Table 1.

**Table 1:** Overview of computational methods for identification of spatially variable genes

Method	Spatial model	Core methodology	Significance test	Input	Gene ranking	Language	Refs
Moran’s I	Graph	Correlation	Permutation	Norm	Moran’s I	Python	[29][30],
Spanve	Graph	Sampling	G-test	Norm	KL divergence	Python	[31]
scGCO	Graph	Graph cuts	CSR model	Norm	FDR	Python	[21]
SpaGCN	Graph	Clustering	Wilcoxon test	Norm	FDR	Python	[22]
SpaGFT	Graph	Fourier transform	Wilcoxon test	Norm	GFT score	Python	[33]
Sepal	Graph	Diffusion model	NA	Norm	Sepal score	Python	[34]
SpatialDE	Kernel	GP	Chi-square	Norm	FSV	Python	[18]
SpatialDE2	Kernel	GP	NA	Norm	FSV	Python	[35]
SPARK	Kernel	GP	Chi-square	Counts	Adj. *p*-value	R	[19]
SPARK-X	Kernel	Covariance test	Chi-square	Counts	Adj. *p*-value	R	[20]
BOOST-GP	Kernel	GP	BFDR	Counts	PPI	R	[37]
GPcounts	Kernel	GP	Chi-square	Counts	LLR	Python	[38]
nnSVG	Graph & Kernel	GP	LR test	Norm	LR statistics	R	[39]
SOMDE	Graph & Kernel	GP	Chi-square	Counts	Adj. *p*-value	Python	[40]

We grouped the methods based on the underlying spatial model. *KL*, Kullback–Leibler; *GP*, Gaussian Process; *FDR*, false discovery rate; *HFRM*, hidden Markov random field; *CSR*, complete spatial randomness, *FSV*, fraction of spatial variance; *BFDR*, Bayesian false discovery rate; *PPI*, posterior probabilities of inclusion; *LR*, likelihood ratio.

### Generating realistic benchmarking datasets for spatial transcriptomics analysis

The primary challenge in benchmarking methods for detecting SVGs is the lack of established datasets with ground truth in real-world tissues and cells. Hence, we focused on devising a strategy to simulate ST data, an approach grounded in precedent studies [19–21, 31, 39]. However, these studies have several limitations regarding the quality of the simulation in recapitulating biological reality and in simulating a diverse set of spatial patterns. Specifically, they often relied on pre-defined spatial clusters and, hence, failed to capture the rich diversity of spatial patterns observed in real biological systems. To address these limitations, we employed the recent scDesign3 framework, significantly advancing the realism and biological relevance of our simulations. Using real data as references, our simulation approach generates more biologically realistic and representative data. Specifically, we modeled the expression of each gene as a function of spatial locations with a GP model. Next, we randomly shuffled the mean parameters to remove spatial correlation between spots, obtaining a non-spatial model for the expression of a given gene. Finally, we synthesized data by mixing the GP and non-spatial model to generate gene expressions with various spatial variability (Fig. 1b).

To account for the diversity of spatial patterns, we collected 50 real-world ST datasets, encompassing nine different ST profiling technologies, 18 tissue types (e.g., heart, brain, kidney, lung), and different tissue of origin and conditions (e.g., healthy and cancer) (Fig. 1c–d; Additional file 1: Fig. S1). Using these datasets as references, we generated simulation data based on the approach described above. For each method, we used these simulated datasets to assess its ranking and classification accuracy based on the Kendall correlation and the area under the Precision-Recall curve (auPRC), respectively (Fig. 1d). Additionally, we examined whether the *p*-values calculated by the methods are statistically calibrated. We also investigated the computational scalability of the methods by measuring memory requirement and running time with different numbers of spots. Importantly, we assessed the impact of SVGs on spatial domain detection analysis, an important downstream task. The results were evaluated based on clustering analysis using the spatial chaos score (CHAOS) metrics, which was originally developed to quantify image segmentation performance in mass spectrometry imaging and here measures the spatial continuity of the detected spatial domains by calculating the average pairwise distance between spots within each cluster [16]. A lower CHAOS score indicates more spatially coherent clusters and thus better spatial domain detection performance*.* We further explored the possibilities of applying these methods to emerging spatial ATAC-seq assays for detecting spatially variable peaks (SVPs). The overall benchmarking metrics are presented in Fig. 1e. Finally, we created a language-agnostic pipeline hosted at the Open Problems in Single-Cell Analysis platform to make our benchmarking framework publicly available to the field (Fig. 1f).

### Comprehensive evaluation of SVG detection methods: accuracy, robustness, and pattern-specific performance

**Fig. 2:**
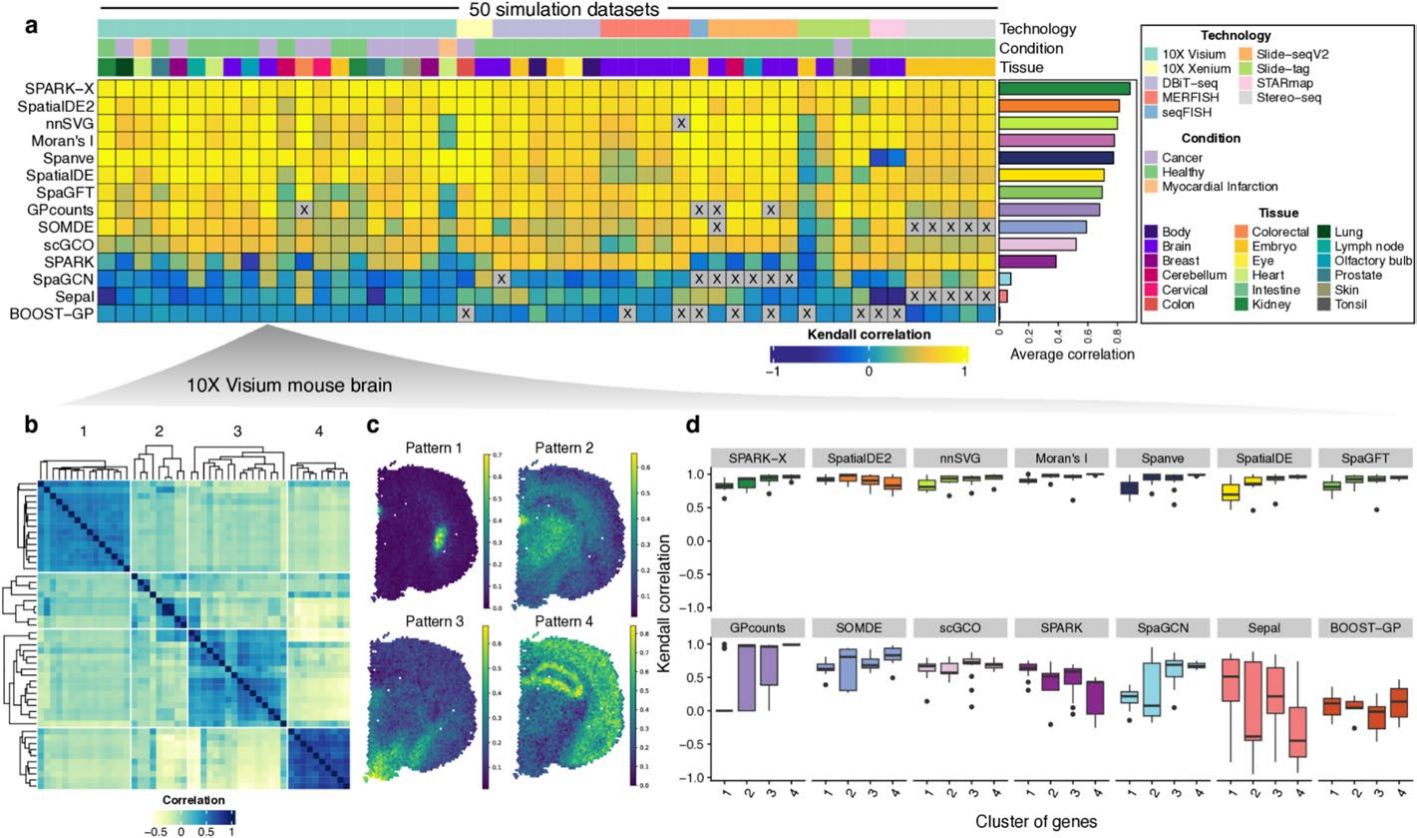
Benchmarking the accuracy using simulated datasets. **a** Heatmap showing the ranking accuracy of each method across all simulated datasets measured by the Kendall correlation. Each row corresponds to a computational method, and each column represents a simulation dataset. A gray color indicates that the method did not produce an output for that dataset. The methods are ordered by their average correlation across all datasets, as shown by the bar plot on the right. The annotations at the top of the heatmap provide details on the spatial technology, tissue type, and biological conditions associated with each dataset. **b** Clustering of genes based on spatial correlation for 10 × Visium data from mouse brain. **c** Visualization of different spatial expression patterns. **d** Boxplot showing the Kendall correlation for each method across different spatial patterns

To rigorously assess the performance of the 14 SVG detection methods, we conducted a multi-faceted analysis using our 50 simulated datasets, focusing on their ability to accurately rank genes based on spatial variation, their computational robustness, and their effectiveness across diverse spatial patterns. Notably, we observed that not all methods could successfully output final results across all the datasets (Fig. 2a). For example, we encountered errors when running SOMDE and Sepal for Stereo-seq datasets and SpaGCN on Slide-seq V2 datasets. Additionally, BOOST-GP could not be completed within a week for three datasets with a high number of spots. These observations underscore the limitations of these methods regarding their numerical stability and computational scalability.

Different methods provide varying scores to rank the genes, reflecting diverse approaches to capturing spatial variability, each with its own strengths in detecting particular types of spatial patterns. For example, *SpatialDE* and *SpatialDE2* use the fraction of spatial variance (FSV) estimated by the GP regression model. In contrast, *SpaGFT* defines a GFT score based on the sum of the low-frequency Fourier coefficients. We assessed if these methods could correctly rank the genes based on the Kendall correlation, which measured the ordinal association between estimated and true spatial variability for each gene. Remarkably, we found that *SPARK-X* consistently outperformed other methods with the highest average correlation (0.88), followed by *SpatialDE2* (average correlation = 0.81) and *nnSVG* (average correlation = 0.8) (Fig. 2a**; **Additional file 2: Table S1). Note that these methods model spatial information using multiple kernel functions (*n* = 11 for *SPARK-X*, *n* = 5 for *SpatialDE2*) with different parameters, which allows for identification of a wide variety of spatial expression patterns. Interestingly, our evaluation revealed that *Moran’s I* statistic, which solely relies on auto-correlation between spots and their neighbors, showed the fourth-best performance with a mean correlation of 0.76, despite its relative simplicity compared to other competitors. We also evaluated if the methods could distinguish SVGs from non-SVGs based on the estimated scores by computing the auPRC, a widely used metric for benchmarking classification tasks. Similarly, we observed that *SPARK-X* showed the highest average auPRC across all datasets (Additional file 1: Fig. S2; Additional file 2: Table S2).

Next, we assessed whether the methods showed different accuracies for different spatial patterns. We selected a 10 × Visium dataset from the mouse brain and clustered the genes by spatial correlation, identifying four major patterns (Fig. 2b–c). We compared the ranking accuracy within each method across the patterns. Interestingly, we found many methods (e.g., *SPARK-X*, *nnSVG*, *Moran’s I*, and *Spanve*) exhibited low accuracy for pattern 1, where genes were highly expressed in a small area and absent elsewhere. Conversely, *SpatialDE2*, *SPARK*, and *Sepal* showed high performance in pattern 1 but low performance in pattern 4. This analysis revealed that different methods display varied performance across distinct spatial patterns. Moreover, these pattern-specific performance differences underscore the importance of considering the expected spatial patterns in a dataset when selecting an SVG detection method and suggest that a combination of methods might be optimal for comprehensive SVG identification.

### Assessing statistical rigor: calibration analysis of SVG detection methods

In addition to providing different scores for ranking genes, the methods (except for *SpatialDE2* and *Sepal*) also determine statistical significance based on distinct statistical tests, allowing users to select SVGs ad hoc. For example, *SpaGCN* and *SpaGFT* use the Wilcoxon test, while the GP-based methods (e.g., *SpatialDE*, *SPARK*, *BOOST-GP*) typically use the Chi-squared test. Notably, *Moran’s I*, implemented by Squidpy, provided three approaches to calculate *p*-values based on the normality assumption of the score test, the permutation test, or the standard normal approximation from permutations [30].

**Fig. 3:**
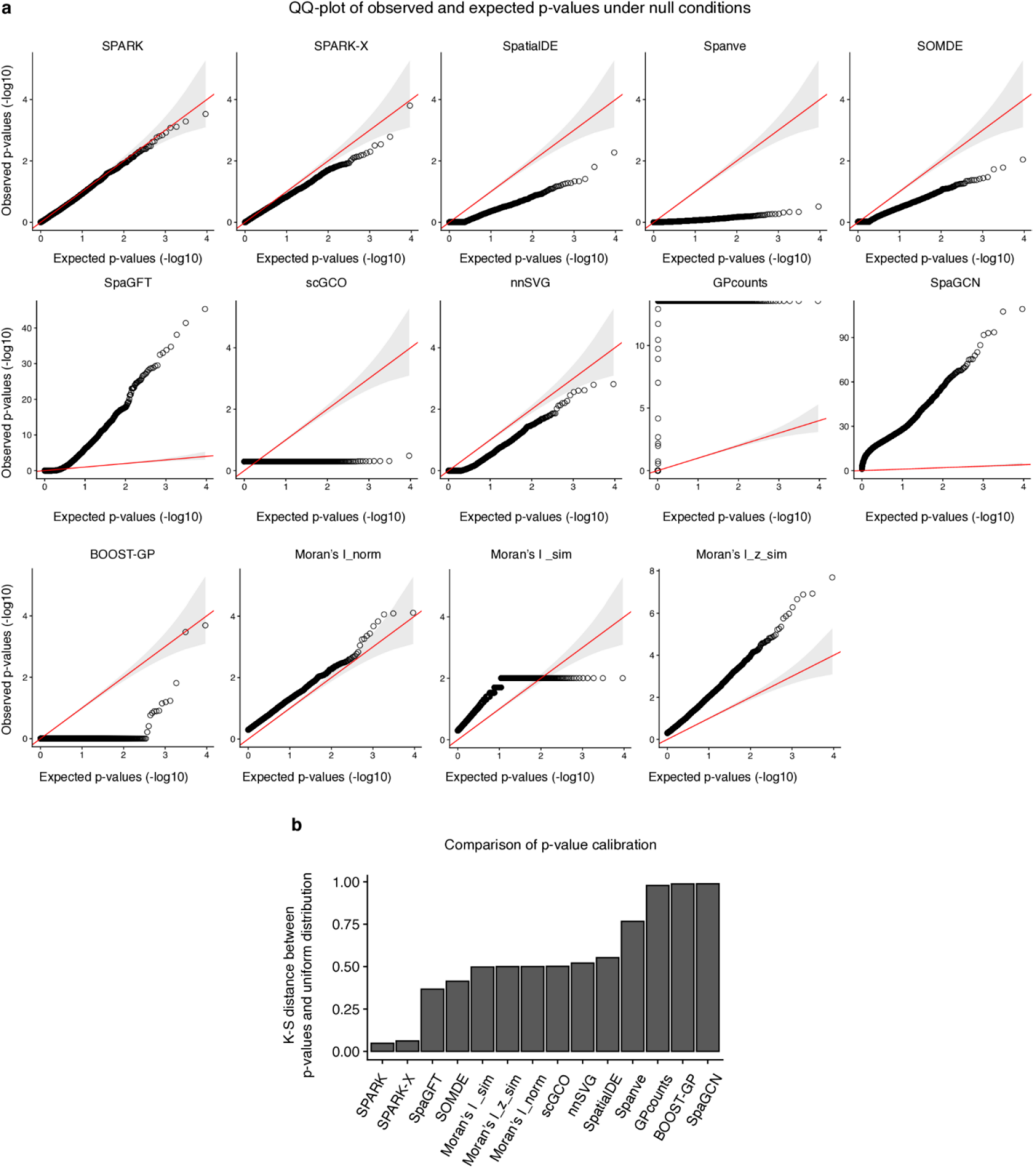
Comparison of statistical calibration of the methods. **a** Quantile–quantile plot of the observed p-values against the expected p-values under the null condition from different approaches. **b** Barplot comparing the K-S distance between observed p-values under null conditions and uniform distribution. A lower value represents a better-calibrated model

On the other hand, Statistical calibration is crucial for the reliable identification of SVG, as it directly impacts the accuracy and interpretability of results. In this section, we evaluated the statistical robustness of the various SVG detection methods, focusing on their ability to produce well-calibrated *p*-values under null conditions, i.e., assuming that gene expression is independent of the spatial location. We used a 10 × Visium mouse olfactory bulk dataset and randomly shuffled the spots to create non-spatially variable expression profiles. A quantile–quantile plot comparing the observed *p*-values against the expected *p*-values showed that *SPARK-X* and *SPARK* produced well-calibrated *p*-values. In contrast, other methods showed poor calibration by producing either overlay liberal *p*-values (too small) or conservative *p*-values (too large) (Fig. 3a). Specifically, six methods (*SpatialDE*, *Spanve*, *SOMDE*, *scGCO*, *nnSVG*, and *BOOST-GP*) generated over-conservative *p*-values, indicating a failure to control type II error. Conversely, four methods (*SpaGFT*, *GPcounts*, *SpaGCN*, and *Moran’s I*) generally overestimated the *p*-values, failing to control type I errors.

To quantitatively compare the statistical calibration across the methods, we measured the Kolmogorov–Smirnov (K–S) distance between the distribution of the observed *p*-values and the uniform distribution. The rationale is that a well-calibrated model should produce uniformly distributed *p*-values between 0 and 1 under the null condition. Therefore, a smaller K–S distance indicates a better-calibrated approach. Our results showed that *SPARK* and *SPARK-X* demonstrated significantly better calibration among the methods, which can be attributed to their use of the recently developed Cauchy *p*-value [41] combination rule to generate final *p*-values from different kernels (Fig. 3b). We performed the same analysis using a 10 × Xenium dataset profiling human colon cancer and observed similar results, again suggesting that besides *SPARK* and *SPARK-X*, most methods were poorly calibrated (Additional file 1: Fig. S3).

### Scalability analysis of SVG detection methods

Next, we evaluated the space and time scalability of the analyzed methods. Given that all methods independently estimate the spatial variability for each gene, the scalability, in theory, is primarily influenced by the number of spatial locations. To benchmark this aspect, we generated ten simulation datasets, each consisting of the same number of genes (*n* = 100) but varying the number of spots, ranging from 100 to 40,000. We applied every method to the ten simulation datasets and recorded the memory consumption and running time as performance metrics (see Methods).

**Fig. 4:**
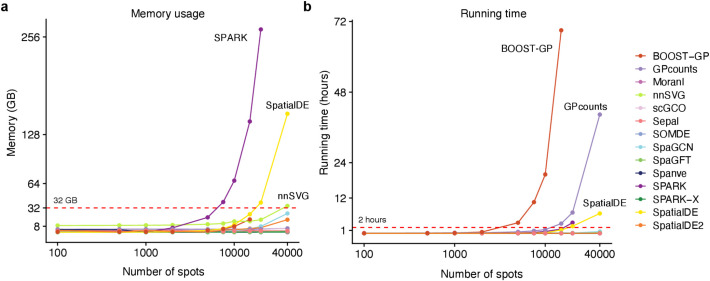
Scalability of the methods. **a** Line plot showing the memory scalability of the methods. The *x*-axis represents the number of spots (log10) of the input datasets with 100 genes. The *y*-axis represents consumed memory (measured as GB) by each method. The red dash Line denotes 32 GB. **b** Same as **a** for time scalability. The *y*-axis represents each method’s consumed time (measured as hours)

Our analysis of memory usage revealed that most methods displayed moderate memory requirements, typically staying below 32 GB, even for datasets with 40,000 spots (Fig. 4a; Additional file 2: Table S3). This finding suggests that these methods can be even executed on modern laptops without encountering memory constraints. Among them, *SOMDE* exhibited the most efficient memory usage across all benchmarking datasets, followed by *Spanve* and *SPARK-X*. In contrast, both *SPARK* and *SpatialDE* exhibited significant increases in memory demand as the number of spots in the dataset increased. For instance, when applied to a dataset with 20,000 spots, *SPARK* necessitated approximately 250 GB of memory, while *SpatialDE* consumed roughly 150 GB when dealing with a dataset containing 40,000 spots. These high memory requirements can be attributed to their use of Gaussian Process regression, which requires the estimation of a covariance matrix across all spots, leading to cubing scaling with the number of spots.

Regarding running time, we observed that *SOMDE* again achieved the best scalability, closely followed by *SPARK-X* and *scGCO*. Notably, most methods completed their computations within a reasonable timeframe of about 2 h (Fig. 4b), making them suitable for practical usage. Both *BOOST-GP* and *GPcounts* exhibited poor scalability with increasing numbers of spots. For instance, *BOOST-GP*’s computational time escalated significantly, requiring 3 days to process a dataset containing 15,000 spots and failing to produce results within 5 days for datasets with 20,000 and 40,000 spots. GPcounts, despite running on a GPU, still required approximately 40 h to process the largest datasets. In summary, our analysis revealed that *SOMDE* and *SPARK-X* exhibited the most favorable scalability when handling datasets with increasing spots, balancing efficiency in both memory usage and running time. These findings highlight the importance of considering computational efficiency when selecting methods for large-scale spatial transcriptomics studies.

### Benchmarking the impact of identified SVGs on spatial domain detection

One of the important applications of spatially resolved transcriptomics is the identification of tissue or region substructures through clustering analysis. For scRNA-seq data, it is a standard practice to utilize HVGs as features for cell clustering [42]. Here, we evaluated whether using SVGs could similarly benefit spatial domain detection. For this task, we obtained the human dorsolateral prefrontal cortex (DLPFC) dataset, which included 12 samples profiled by the 10 × Genomics Visium platform [43]. Each sample was manually annotated as one of the six prefrontal cortex layers (L1-6) and white matter (WM). We also obtained 12 samples from HPV-negative oral squamous cell carcinoma (OSCC) [44] and 8 samples from HER2-positive breast tumors (HER2) [45]. Both datasets were annotated by expert pathologists based on tissue morphology. Visualization of these datasets with ground truth are available in Additional file 1: Fig. S4.

**Fig. 5:**
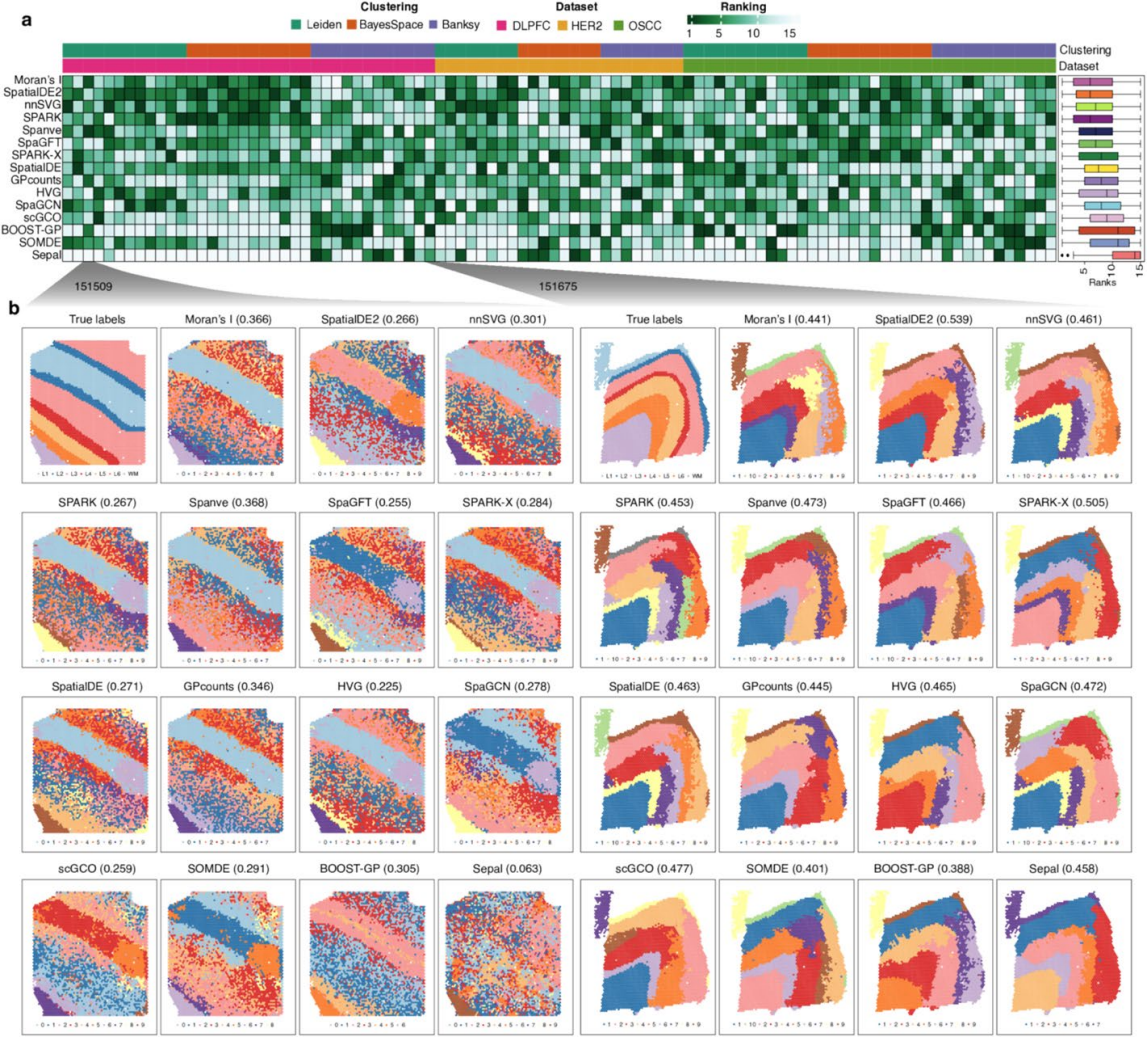
Impact of detected SVGs on spatial domain detection analysis. **a** Heatmap showing the clustering performance as evaluated using ARI. Each row represents a benchmarked SVG detection method, and each column represents a sample clustered by an algorithm. The colors refer to rank of the SVG detection method within the corresponding sample-clustering method combination. **b** Left: Visualization of true labels for sample 151,509 and the clustering results using the Leiden algorithm based on SVGs from the benchmarked methods. ARIs are shown on the top.Right: visualization forsample 151,675 using the Banksy algorithm for clustering

We next applied the 14 SVG detection methods to these 32 samples and selected the top 2000 SVGs for each sample based on the estimated scores by each method. To establish a baseline for comparison, we also used the top 2000 HVGs identified by scanpy. Next, we clustered the spots using three different clustering algorithms, including Leiden (resolution = 1), BayesSpace [46], and Banksy [47]. Of note, BayesSpace and Banksy were specifically designed for spatial transcriptomics data by leveraging spatial information. The clustering results were evaluated against the annotated spatial domains using the Adjusted Rand Index (ARI), which measures the similarity between two data clustering results of the same data. To ensure a fair comparison across diverse samples and clustering methods, we computed ranks of the SVG detection methods within each sample-clustering method combination based on their ARI scores (Fig. 5; Additional file 2: Table S4). Notably, we observed that most SVG detection methods consistently improved spatial clustering accuracy relative to HVG-based feature selection, underlying the value of incorporating spatial information to gene selection for this specific analysis. Only a few methods (*SpaGCN*, *scGCO*, *BOOST-GP*, *SOMDE*, and *Sepal*) failed to outperform HVGs in this benchmarking, which indicates potential limitations in their sensitivity across tissue architectures. Interestingly, Moran’s I achieved the best performance with a mean rank of 6.5, closely followed by SpatialDE2 (mean rank = 6.6) and nnSVG (mean rank = 6.8), showcasing their effectiveness in capturing informative genes for identifying spatial domains. Overall, these findings underscore the utility of SVG-based feature selection for spatial domain detection and reveal that simpler statistical approaches like Moran’s I can be highly effective in practice.

### Benchmarking SVG detection methods with spatial ATAC-seq profiles

Recent technological advances have allowed for profiling spatially-resolved chromatin accessibility [48, 49]. However, specific methods for detecting spatially variable open chromatin regions (i.e., spatially variable peaks, abbreviated as SVPs) are currently lacking. In this section, we aimed to investigate the feasibility of applying methods developed for SVG detection to analyze spatial chromatin accessibility profiles. For this, we downloaded spatial ATAC-seq data from mouse gestational development at embryonic days of E12.5, E13.5, and E15.5 [49]. Following data processing, we obtained a cell-by-peak matrix for each of the samples and applied the methods to detect SVPs. However, given that these methods were not specifically designed for this task, we encountered several challenges. For example, *BOOST-GP* and *GPcounts* failed to produce results even after 120 h of running because the number of peaks substantially exceeded the number of genes, highlighting the limitation of these two methods in terms of scalability. Additionally, *SPARK* encountered memory issues and did not yield any results.

**Fig. 6:**
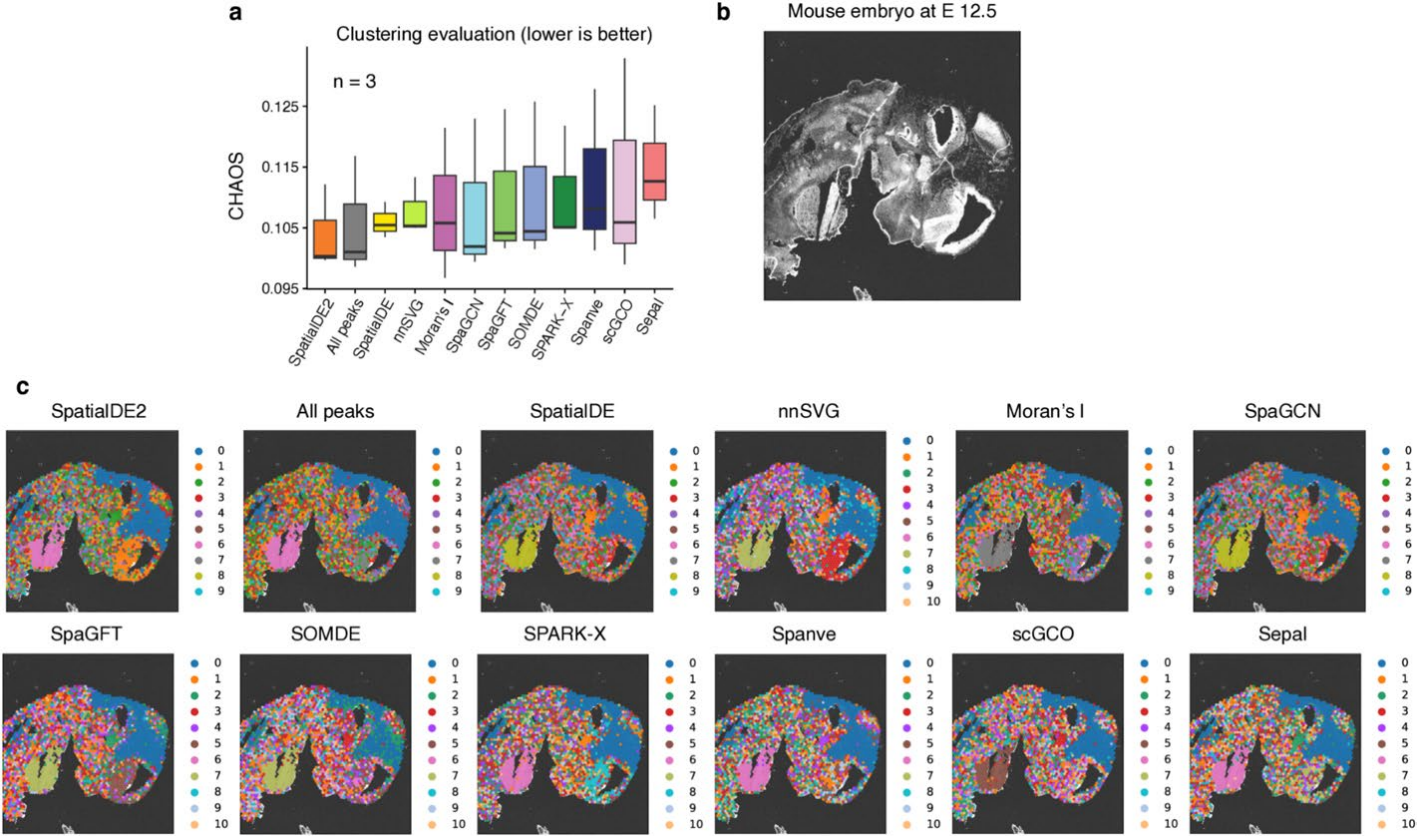
Benchmarking the methods on spatial ATAC-seq data. **a** Boxplot showing the spatial CHAOS score across different methods. A lower value indicates a better performance for spatial clustering. **b** Image of a mouse embryo at days of E12.5. **c** Visualization of obtained clusters using spatially variable peaks identified by different methods

As in the previous section, we wanted to investigate if SVPs recovered from these procedures could boost spatial clustering. We selected the top 20,000 peaks for each method and used Leiden-based clustering analysis to group the spots and evaluate the quality of the SVPs. Because the ground truth is unavailable in this dataset, we measured the spatial continuity and locality of the clusters using the spatial chaos score (CHAOS) [16]. The underlying assumption is that a more accurate identification of SVPs would yield more continuous and cohesive clusters [16]. We also included the results generated using all the peaks as a baseline. Interestingly, we observed that *SpatialDE2* outperformed other methods (mean CHAOS = 0.104), indicating that it has the potential to identify biologically meaningful SVPs (Fig. 6a–c; Additional file 1: Fig. S5). Moreover, our analysis revealed that using all peaks yielded the second-best performance (mean CHAOS = 0.105). This finding suggests that more specialized methods are required to analyze spatial chromatin accessibility data. Such methods would be invaluable for advancing our understanding of spatial gene regulation and chromatin dynamics in complex tissues.

### Overall performance of SVG detection methods

**Fig. 7:**
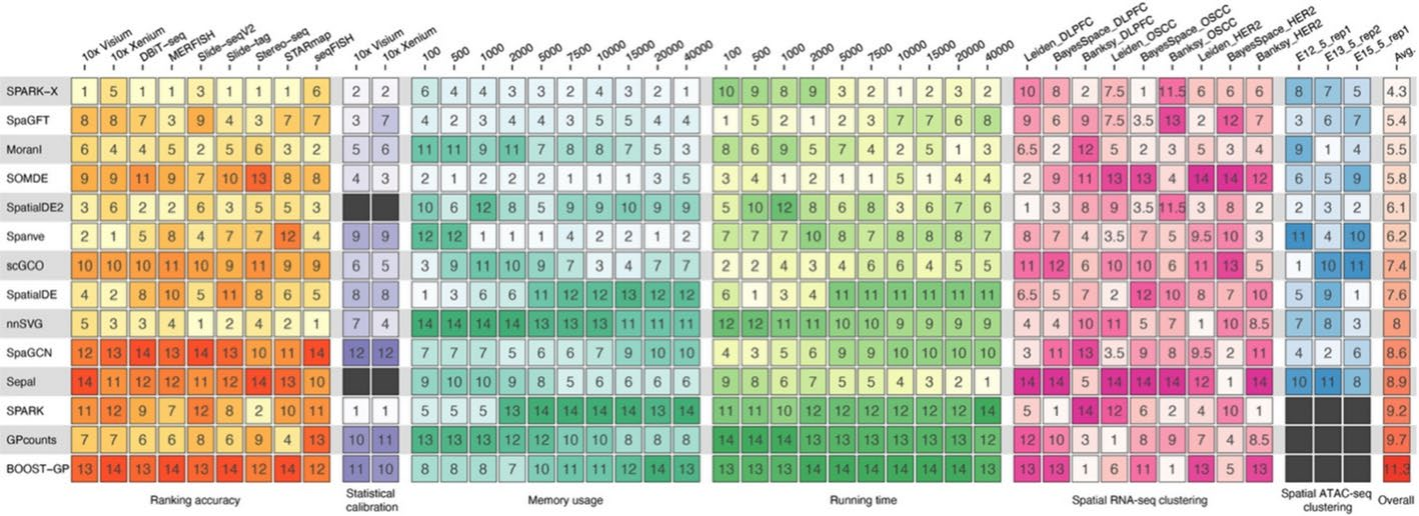
Overall performance. Heatmap summarizing the overall performance of the methods in terms of their ability to accurately rank genes based on estimated spatial variation, statistical calibration of p-values under null conditions, memory usage and running time requirement for different dataset sizes, impact on clustering of spatial RNA-seq and ATAC-seq data. The colors and numbers denote the ranking of each method for each specific metric, with the average rank shown on the right. The black colors indicate that the results are not available

Finally, we summarized our benchmarking results by comparing the methods across various metrics, including gene ranking accuracy, statistical calibration, memory usage, and running time scalability, and their impact on spatial RNA-seq and ATAC-seq clustering. For ranking accuracy, we evaluated the methods for each spatial technology using the average Kendall correlation. Statistical calibration was assessed by ranking the methods based on the K-S distance between the observed and expected *p*-values under the null hypothesis. Scalability was evaluated by ranking the methods for each dataset based on memory usage and running time with different numbers of spots. For spatial RNA-seq clustering, methods were ranked for each clustering algorithm (Leiden, BayesSpace, and Banksy) and dataset (DLPFC, OSCC, and HER2) combination using the mean rank of ARI values across the samples. For spatial ATAC-seq clustering, methods were ranked for each dataset based on the CHAOS score. To determine overall performance, we calculated the average ranking for each method across all these metrics (Fig. 7).

Our analysis revealed *SPARK-X* as the top-performing method, with an average ranking of 4.3. It demonstrated the best performance in correctly ranking genes based on estimated spatial variation for six out of nine ST profiling techniques, showcasing its robust performance. Additionally, *SPARK-X* produced well-calibrated *p*-values and exhibited second-best scalability for large-scale ST datasets (e.g., > 20,000 spots), mostly attributed to the fact that it directly compared the expression and spatial distance covariance matrix rather than using GP regression to estimate spatial variation, unlike its predecessor *SPARK*. *SpaGFT* ranked the second best with an average ranking of 5.4, performing well in ranking accuracy, statistical calibration, and computation scalability. Unlike *SPARK-X,* which uses multiple kernel functions to model spatial patterns, *SpaGFT* represents the spatial data as a graph and employs the Fourier transform to estimate spatial variation. Surprisingly, *Moran’s I*, a simple method based on autocorrelation between spots and their spatial neighbors, achieved the third-best performance. This method demonstrated a good gene ranking ability and competitive computational efficiency, notably outperforming other methods in spatial domain detection. The strong performance of this classic metric, which has been largely overlooked in recent benchmarking [26] and methodology development efforts [19, 20, 22, 33, 34, 38–40], suggests that it should be included as a baseline in future studies.

### Guideline for method selection

Our comprehensive benchmarking of 14 computational methods revealed that no single approach consistently outperforms others across all evaluation metrics. As such, method selection for detecting SVGs should be tailored to the specific goals and constraints of a study. Below, we provide recommendations based on usability, performance, scalability, and downstream utility.

In practice, usability is a key factor in method adoption. Among the evaluated tools, only a few Python-based methods, *Moran’s I*, *Sepal*, and *Spanve*, can be directly applied to the AnnData object, a widely used data structure in the Python-based single-cell analysis ecosystem. Of these, *Moran’s I*, implemented within the Squidpy framework, stands out as the most accessible and user-friendly. By contrast, R-based tools such as *BOOST-GP*, *nnSVG*, *SPARK*, and *SPARK-X* require custom input formats or additional preprocessing steps, making them less convenient to integrate into R-based existing analysis tool (e.g., Seurat).

If the primary goal is to rank genes based on spatial variability to prioritize candidate regulators or inform downstream functional studies, methods employing multi-kernel Gaussian Process models consistently achieve superior performance. In particular, *SPARK-X* demonstrated the highest overall ranking accuracy across diverse datasets and spatial patterns (Fig. 2a). For more exploratory analyses, *Moran’s I* offers a lightweight alternative with competitive accuracy. Therefore, we recommend *SPARK-X* for comprehensive analyses, and *Moran’s I* for preliminary analysis.

Regarding scalability, our benchmarking on datasets containing up to 40,000 spatial spots revealed that *SOMDE* is the most computationally efficient method, exhibiting low memory usage and fast runtime (Fig. 4). *SPARK-X* also performed well, achieving a strong balance between accuracy and scalability by avoiding the cubic computational burden associated with Gaussian Process regression. Accordingly, we recommend *SOMDE* or *SPARK-X* for high-throughput spatial transcriptomics applications where computational resources and memory are a limiting factor.

When performing spatial domain detection, the choice of method significantly influences clustering performance. Our analysis using DLPFC, OSCC, and HER2 tumor datasets showed that most SVG-based selections improved clustering over HVGs, and *Moran’s I* achieved the best overall performance followed by *SpatialDE2* and *nnSVG* (Fig. 5). These methods were particularly effective in identifying informative features that capture tissue architecture. Therefore, for analyses focused on spatial domain detection, especially in histologically structured tissues, we recommend using *Moran’s I*, *SpatialDE2*, or *nnSVG*.

Finally, our evaluation of statistical calibration revealed that most methods produced poorly calibrated *p*-values, meaning they tended to systematically under- or overestimate the true statistical significance of spatial variability (Fig. 3). Such miscalibration can result in inflated false positive rates or missed true spatial signals, undermining the reliability of downstream analyses. To mitigate this issue, we recommend selecting SVGs based on a fixed ranking threshold (e.g., the top 2000 genes by score) rather than relying on significance.

## Discussion

Over a dozen computational methods have recently been developed to identify spatially variable genes (SVGs) in spatial transcriptomics data. These methods diverge substantially in several aspects, including the assumptions in modeling spatial relationships between cells (graph vs. kernel); the algorithms to estimate spatial variation (e.g., auto-correlation vs. Gaussian Process regression vs. graph cut); the statistical tests to determine significances (e.g., permutation test vs. Wilcoxon test vs. Chi-square test); the choice of input data (raw counts vs. normalized data); and the programming languages (Python vs. R) (Table 1). These differences complicate the user selection process, further exacerbated by the lack of realistic benchmarking to evaluate the performance of these methods.

A major challenge in SVG evaluation is the lack of gold-standard datasets. Previous studies often used simulated data by generating non-SVG profiles, which oversimplifies the distinction between SVGs and non-SVGs into a binary classification. Since spatial variability is a continuous measure, evaluating results within a binary framework is highly problematic. To address this, we proposed a novel strategy using scDesign3 and real-world spatial transcriptomics data to create biologically realistic datasets with varying degrees of spatial variation. This simulation and evaluation approach represents an advancement in realistic modeling and more accurate benchmarking of spatial transcriptomics data. However, our approach has certain limitations and opens up new avenues for future research. For instance, the strategy requires pre-selection of genes with high spatial variation, which could introduce biases favoring certain methods. Additionally, our evaluation computes the Kendall rank correlation within each gene, which does not directly compare methods for detecting different spatial patterns. Future research should refine these approaches and further enhance the robustness and applicability of benchmarking methods in spatial transcriptomics.

Another limitation of our current benchmarking framework is that we did not evaluate the rotation invariance properties of these SVG detection methods or assess their sensitivity to spatial orientation. In practice, tissue positioning during sample preparation can vary considerably, and the same biological tissue may be oriented differently across experiments or datasets. Future benchmarking efforts should therefore investigate whether SVG detection methods maintain consistent performance regardless of tissue rotation or spatial transformation.

We demonstrated that using SVGs generally improved spatial domain detection compared to relying solely on HVGs. This was shown through different clustering methods applied to three distinct datasets with well annotated domains. These findings underscore the importance of incorporating spatial information, which enhances clustering analysis by providing a more comprehensive understanding of the architecture of complex tissues. As novel technologies like Slide-Tags [50] emerge, enabling the simultaneous acquisition of single-cell measurements and spatial data, we anticipate a surge in the adoption and popularity of SVG identification tools in various downstream analysis tasks.

Compared to transcriptomic data, spatial ATAC-seq data present unique and substantial analytical challenges that our benchmarking empirically confirms, making the identification of spatially variable peaks a difficult and unresolved problem. In our benchmarking, we found that using all peaks led to better clustering performance than most methods, as measured by the CHAOS score. This surprising result likely reflects several inherent limitations of spatial ATAC-seq data: (i) the data are extremely sparse, with a large proportion of peaks exhibiting zero counts across most spatial locations, which hampers reliable estimation of spatial variability; (ii) the signal is often binary or near-binary, contrasting sharply with the continuous and over dispersed nature of RNA-seq data, and thus requires fundamentally different statistical modeling; and (iii) the high dimensionality of peak matrices (20,000–70,000 peaks compared to typical gene counts of 2000–5000) increases the risk of overfitting and further complicates model inference. Indeed, these computational challenges were evident in our benchmarking: BOOST-GP and GPcounts failed to complete even after 120 h, while *SPARK* encountered memory failures.

Collectively, these characteristics may underlie the poor performance of current methods when applied to spatial chromatin accessibility data. Moving forward, there is a pressing need to develop new algorithms specifically tailored to the sparsity, distributional properties, and data structure of spatial ATAC-seq to fully leverage its potential in dissecting spatial epigenomic regulation.

While our study focuses on spatial transcriptomics and spatial ATAC-seq, other spatially resolved omics data, such as spatial proteomics, are also emerging. To interpret biological activity within spatial contexts for various modalities—such as genes, proteins, peaks, and motifs—specialized approaches and dedicated gold-standard datasets are needed. Our study showed that some methods could be applied to spatial ATAC-seq, aiding in the identification of potential SVPs. We refer to them as “potential” due to the absence of ground truth data. Instead, we utilized SVPs for clustering and evaluated the spatial locality and continuity of the resulting clusters. It is important to note that not all methods were capable of detecting SVPs due to limitations in memory or algorithmic complexity. Tools focused on discerning SVPs can potentially reveal the regulatory elements that govern gene expression profiles within specific spatial sub-regions. This, in turn, can enhance our understanding of the regulatory mechanisms governing SVGs and, consequently, the spatial organization of tissues and tumors. In the future, integrating SVGs and SVPs through novel algorithms holds tremendous potential to facilitate the construction of accurate spatially aware gene regulatory networks.

Compared to previous benchmarking studies [23–25], our work introduces several important innovations that distinguish it in both scope and methodological rigor. First, while prior benchmarks primarily treated SVG detection as a binary classification task, we formulated it as a gene ranking problem, which better reflects how existing methods are applied in practice. Based on this formulation, we implemented a novel simulation framework using scDesign3 that generates gene-level spatial variability derived from real-world data. This allowed us to more accurately assess the ability of each method to rank genes by true spatial variability across a continuum, rather than relying on artificial or binary ground truths. Second, our study includes more computational methods (*n* = 14) and covers a wider range of spatial transcriptomics technologies, spanning 50 datasets across 9 platforms, compared to ≤ 7 methods in prior studies. As a result, to our knowledge, this represents the largest and most comprehensive benchmarking effort to date for SVG detection.

Despite these differences in scope and design, we observed several consistent findings with prior studies. For example, both our study and previous benchmarks identified *SPARK-X* and *nnSVG* as top-performing methods in terms of sensitivity and specificity. In addition, through quantitative evaluation, we found that *p*-value estimates produced by most methods tend to be poorly calibrated, which was also observed in prior works and represents a critical limitation for biological interpretation. However, our broader evaluation also revealed additional insights not captured in earlier work. Notably, we found that *Moran’s I*, a simple spatial autocorrelation statistic, demonstrated competitive performance across a wide range of datasets and downstream tasks, particularly in spatial domain detection. This is the first benchmarking study to identify *Moran’s I* as a top performer, suggesting that this method may have been underappreciated in prior evaluations. Our findings also revealed the need for specialized methods for spatial ATAC-seq data, as most existing approaches showed poor performance on chromatin accessibility profiles.

A lack of standardized evaluation workflow often leads to different assessment results of the same methods [51]. To overcome this issue, we created a living benchmarking pipeline for the community to allow for continuously integrating new methods, datasets, and metrics (https://github.com/openproblems-bio/task_spatially_variable_genes). We envision that this will drive the development of more robust and accurate methods for SVG detection. Taken together, our study provides a detailed evaluation of various SVG detection methods across simulated and real-world datasets of spatial transcriptomics and spatial-ATAC-seq.

## Conclusions

In this study, we performed a comprehensive benchmarking of computational methods for identifying spatially variable genes (SVGs) and peaks (SVPs) across a wide range of spatial omics platforms. By reframing SVG detection as a gene-ranking task and leveraging biologically realistic simulations generated with scDesign3, we established a framework that better reflects real-world analysis workflows compared to approaches. Our results revealed substantial differences in method performance, driven by divergent statistical assumptions and algorithmic designs. Notably, we found that simple statistical approaches, including Moran’s I, can perform competitively with more complex methods and may be underutilized in current workflows. Our evaluation also exposed major limitations of existing methods when applied to spatial ATAC-seq data, underscoring the pressing need for algorithms specifically tailored to the sparsity and binary nature of chromatin accessibility profiles. To facilitate ongoing development and reproducibility, we provide an open and extensible benchmarking framework capable of integrating new methods, datasets, and evaluation metrics. As spatial omics technologies continue to evolve, the joint analysis of SVGs, SVPs, and other spatially variable molecular features will be critical for building accurate, spatially resolved models of gene regulation and tissue organization.

## Methods

### Simulation of biologically realistic spatial data with various spatial variability

We used scDesign3 to generate biologically realistic data with various spatial variability using real-world spatial transcriptomics data as a reference. Specifically, we modeled the marginal distribution of expression for each gene using the function *fit_marginal (mu_formula* = “s(spatial1, spatial2, bs = ‘gp,’ k = 500),” sigma_formula = “1,” family_use = “nb”) which fitted the data with a generalized GP model under the Negative Binomial distribution. The joint distribution of genes was modeled using the function fit_copula (family_use = “nb,” copula = “gaussian”). Next, we extracted the mean parameters for each gene across all spots, denoted by ${\mu }_{s}(s)$*,* and randomly shuffled the parameters to remove spatial correlation between the spots, generating a non-spatially variable mean function ${\mu }_{ns}(s)$. We used the function *simu_new* to generate simulation data as follows:

$$\mu (s) = \alpha \cdot {\mu }_{s}(s) + (1 - \alpha ) \cdot {u}_{ns}(s)$$

where $\alpha$ denotes the fraction of spatial variability in simulated gene expression. When $\alpha =0$, the expression was sampled from a randomly shuffled distribution without spatial correlation (i.e., non-spatially variable genes). while for $\alpha =1$, the expressions were generated from the GP model with the same spatial variability as the reference data. By varying $\alpha$, we were able to generate biologically realistic data with various spatial variability for each gene. We used 21 different values for $\alpha$ between 0 and 1 for simulation.

To account for the diversity of spatial data and patterns, we collected a total of 50 real-world datasets across 17 different tissue types and nine technologies, including 10 × Visium (*n* = 20), Slide-seqV2 (*n* = 5), Slide-tag (*n* = 4), DBiT-seq (*n* = 6), Stereo-seq (*n* = 5), 10 × Xenium (*n* = 2), MERFISH (*n* = 5), seqFISH (*n* = 1), and STARmap (*n* = 2). We filtered mitochondrial and lowly expressed genes for each dataset, and generated a corresponding simulation dataset using the above framework. We used these datasets to evaluate the performance of the methods.

### Simulation of spatial data with different number of spots

To evaluate the computational scalability of the methods with the number of spatial spots, we first defined a covariance matrix $K\in {R}^{m\times m}$ as follows:

$$K(a, b)={\sum }_{n=1}^{N}{\beta }_{n}\cdot \text{exp}\left(\frac{||x(a) - x(b)|{|}^{2}}{2\cdot {{l}_{n}}^{2}}\right)$$

$$({\beta }_{1}, {\beta }_{2},\cdots , {\beta }_{N}) \sim Dirichlet(1/N,\cdots , 1/N)$$

where $m$ is the number of spots, $x(a)$ and $x(b)$ denote two spatial locations, $N$ is the number of kernels, ${\beta }_{n}$ is the weight of the $n$ th kernel and is sampled from a Dirichlet distribution, and ${l}_{n}$ denotes the length scale. By sampling $\beta$ for each gene, we obtained different spatial covariance matrices, thus creating different spatial patterns. We next sampled expression ${\lambda }_{j} \in {R}^{m}$ for a gene $j$ across all locations from a multivariate normal distribution (MVN) as follows:

$$log({\lambda }_{j}) \sim MVN(\mu , K)$$

Because some methods can only work on raw counts, we next converted the data to counts as follows:

$${{\lambda }_{ij}}{\prime} = \frac{{\lambda }_{ij}}{\sum_{j=1}{\lambda }_{ij}}$$

$${y}_{ij} \sim Poisson(s\cdot {{\lambda }_{ij}}{\prime} )$$

where $s$ denotes the Library size and is set to 10,000 for all locations. We generated ten simulation datasets as described above. Each dataset had the same number of genes (*n* = 100) and a different number of spots (*n* = 100, 500, 1000, 2000, 5000, 7500, 10,000, 15,000, 20,000, 40,000).

### Human dorsolateral prefrontal cortex (DLPFC) datasets

We downloaded human dorsolateral prefrontal cortex (DLPFC) spatial transcriptomics data from https://research.libd.org/spatialLIBD/. This included 12 samples profiled by the 10 × Genomics Visium platform [43]. Each sample was manually annotated as one of the six prefrontal cortex layers (L1-6) and white matter (WM). This dataset has been commonly used to evaluate the algorithms for spatial clustering and domain detection analysis in the field [46, 47, 52]. In this study, we used these datasets to test the impact of detected SVGs on spatial domain detection analysis.

### Identify SVGs with computational methods

Below, we describe the details of running the methods to identify SVGs.

#### Moran’s I

* Moran’s I* measures the correlation of gene expression between a spatial location and its neighbors [29]. We identified spatial neighbors using the function *spatial_neighbors* and computed the *Moran’s I* score using the function *spatial_autocorr (n_perms* = *100)* from Squidpy (v1.2.3) [30].

#### Spanve

*Spanve* (Spatial Neighborhood Variably Expressed Genes) is a non-parametric statistical approach for detecting SVGs [31]. Similar to *Moran’s I*, this method uses the difference between a location and its spatial neighbors to estimate the spatial variation. Specifically, for each gene, it computes Kullback–Leibler divergence between space-based and randomly sampled expressions. The significance is calculated by the G-test. We installed *Spanve* (v0.1.0) and ran the method by following the tutorial: https://github.com/zjupgx/Spanve/blob/main/tutorial.ipynb.

#### SpaGFT

*SpaGFT* is a hypothesis-free Fourier transform model to identify SVGs [33]. It decomposed the signal from the spatial domain to the frequency domain based on a spatial KNN graph and estimated a GFTscore per gene on the Fourier coefficient for low-frequency signals. We installed *SpaGFT* (v0.1.1.4) and ran it by following the tutorial: https://spagft.readthedocs.io/en/latest.

#### SpaGCN

*SpaGCN* is a graph convolutional network (GCN)-based approach that integrates gene expression, spatial location, and histology to identify SVGs [22]. It first identifies spatial domains through clustering and then detects SVGs that are enriched in each domain. We installed *SpaGCN* (v1.2.5) and ran the method by following the tutorial: https://github.com/jianhuupenn/SpaGCN/blob/master/tutorial/tutorial.ipynb.

#### scGCO

*scGCO* (single-cell graph cuts optimization) utilizes a hidden Markov random field (HMRF) with graph cuts to identify SVGs [21]. We installed *scGCO* (v1.1.0) and executed the method by following the tutorial: https://github.com/WangPeng-Lab/scGCO/blob/master/code/Tutorial/scGCO_tutorial.ipynb.

#### Sepal

*Sepal* assesses the degree of randomness exhibited by the expression profile of each gene through a diffusion process and ranks the genes accordingly [34]. We computed the *Sepal* score using Squidpy (v1.2.3) by following the tutorial: https://squidpy.readthedocs.io/en/stable/auto_examples/graph/compute_sepal.html.

#### SpatialDE

*SpatialDE* is one of the pioneer methods for identifying SVGs [18]. It models the normalized spatial gene expression using the Gaussian process regression and estimates the significance by comparing the models with and without spatial covariance. We installed *SpatialDE* ~ (v1.1.3) with pip and processed the data with the functions NaiveDE.stabilize and NaiveDE.regress_out. The results were obtained by running the function SpatialDE.run.

#### SpatialDE2

*SpatialDE2* is a flexible framework for modeling spatial transcriptomics data that refines *SpatialDE* by providing technical innovations and computational speedups [35]. We obtained the source code from https://github.com/PMBio/SpatialDE and estimated spatial variance using the function SpatialDE.fit.

#### SPARK

*SPARK* extended the computation framework proposed in SpatialDE by directly modeling the raw count data using a generalized linear spatial model (GLSM) based on Poisson distribution [19]. We obtained *SPARK* (v1.1.1) from https://github.com/xzhoulab/SPARK and ran the method by following the tutorial https://xzhoulab.github.io/SPARK/02_SPARK_Example.

#### SPARK-X

*SPARK-X* is a non-parametric covariance test method based on multiple spatial kernels for modeling sparse count data from spatial transcriptomic experiments [20]. We ran *SPARK-X* (v1.1.1) by following the tutorial: https://xzhoulab.github.io/SPARK/02_SPARK_Example.

#### BOOST-GP

* BOOST-GP* is a Bayesian hierarchical model to analyze spatial transcriptomics data based on zero-inflated negative binomial distribution and Gaussian process [37]. We downloaded the source codes of *BOOST-SP* from https://github.com/Minzhe/BOOST-GP and ran the function boost.gp by setting the parameters iter to 100 and burn to 50.

#### GPcounts

* GPcounts* implemented Gaussian process regression for modeling counts data using a negative binomial likelihood function [38]. We obtained the source codes of *GPcounts* from https://github.com/ManchesterBioinference/GPcounts and followed the tutorial https://github.com/ManchesterBioinference/GPcounts/blob/master/demo_notebooks/GPcounts_spatial.ipynb. We ranked the genes by the log-likelihood ratio (LLR), representing the ratio between the dynamic and constant (null) models. We noted that *GPcounts* sometimes failed to generate results for certain genes. In this case, we set the LLR as 0.

#### nnSVG 

*nnSVG* is a method built on nearest-neighbor Gaussian processes to identify SVGs [39]. We installed the package (v1.2.0) from Bioconductor and ran the method by following the tutorial https://bioconductor.org/packages/release/bioc/vignettes/nnSVG/inst/doc/nnSVG.html.

SOMDE

#### SOMDE

* SOMDE* uses a self-organizing map (SOM) to cluster neighboring locations into nodes and then uses a Gaussian process to fit the node-level spatial gene expression to identify SVGs [40]. We installed *SOMDE* (v0.1.7) and followed the tutorial https://github.com/WhirlFirst/somde/blob/master/slide_seq0819_11_SOM.ipynb to run the method.

### Benchmarking accuracy for ranking and classification

We applied each method to the simulated datasets to rank the genes. Next, we calculated the ranking accuracy based on the Kendall rank correlation for each gene using the estimated and true spatial variability as follows:

$$\uptau = \frac{(\text{number of concordant pairs}) - (\text{number of discordant pairs})}{\text{number of pairs}}$$

The correlation has a maximum value of 1 if the two ranks perfectly agree with each other, and otherwise, a minimum value of − 1. To test whether the methods can distinguish spatially vs. non-spatially variable genes, we also computed the classification accuracy based on the area under the precision-recall curve (auPRC) using the function pr.curve from the R package PRROC [53].

### Benchmarking statistical calibration

We used 10 × Visium data generated from mouse olfactory bulb consisting of 1185 spatial spots and to assess the statistical calibration, in line with previous study [19]. We filtered the genes by the number of expressed spots (*n* = 500). We permuted the spots to obtain non-spatially variable expressions. We applied each method to this permuted data and obtained the estimated *p*-values under null conditions. Note that SpatialDE2 and Sepal were excluded from this evaluation because they do not estimate statistical significance. We then plotted the Q-Q plot between expected and observed *p*-values to visualize the calibration. To quantitatively compare the statistical calibration between different methods, we calculated the Kolmogorob–Smirnov (K–S) distance between observed *p*-values and uniform distribution from 0 to 1. A lower distance indicates a better-calibrated estimation of *p*-values.

### Benchmarking scalability with the number of spatial spots

We used the Snakemake [54] workflow (v7.25.2) management system to evaluate the scalability of each method with the number of spatial spots. We ran each method on a dedicated HPC node with an AMD EPYC 7H12 64-core Processor using the same computational resource (1TB memory, 120 h, and 10 CPUs) defined by the Snakemake pipeline. For methods (i.e., *GPcounts* and *SpatialDE2*) that require a graphics processing unit (GPU) for running, we used an A100 with 40GB of memory. We measured the memory usage and running time using the benchmark directive provided by the Snakemake tool (–benchmark). We could not run *SPARK* for datasets with 40,000 spots because of memory issues. Moreover, *BOOST-GP* did not generate results for datasets with 20,000 and 40,000 spots within 120 h.

### Benchmarking the impact of identified SVGs on spatial domain detection analysis

We utilized the Human DLPFC datasets to evaluate the impact of identified SVGs on spatial domain detection. For ran the methods and selected the top 2000 SVGs based on the ranking (see Table 1). The HVGs were identified using the function scanpy.pp.highly_variable_genes, and the top 2000 genes were used as our baseline for comparison. We used these genes as features for clustering with the following algorithms.

Leiden. We used the function scanpy.tl.pca with the default parameters to perform dimension reduction and the function scanpy.pp.neighbors to generate a k-nearest-neighbor graph. The spots were clustered by the function scanpy.tl.leiden (resolution = 1).

BayesSpace. BayesSpace is a tool designed for clustering and enhancing the resolution of spatial gene expression profiles 46. We installed BayesSpace (v1.1.4) from Bioconductor and followed the tutorial (https://www.ezstatconsulting.com/BayesSpace/articles/maynard_DLPFC.html) to run BayesSpace by using different features as input.

Banksy. Banksy is an algorithm that embeds cells in a product space of their own and the local neighborhood. We obtained the Banksy R package (v0.99.12) from https://github.com/prabhakarlab/Banksy and executed it with the default setting. Specifically, the counts were normalized using the function NormalizeData from Seurat. The matrix was subset by only keeping the SVGs as identified by each method. Next, we computed the component neighborhood matrices using the function computeBanksy. We then ran the functions runBanksyPCA and clusterBanksy with the default settings to obtain final clustering results.

After clustering the data using different features and the above algorithms, we compared the obtained clusters with the annotated layers using Adjusted Rand Index (ARI). A higher ARI indicates a better clustering result.

### Benchmarking the methods for spatial ATAC-seq data

We downloaded spatial ATAC-seq data of mouse embryos at stages E12.5, E13.5, and E15.5 from GEO with accession number GSE214991. We first identified open chromatin regions by peak calling on all the spots using MACS2[55] (–nomodel–nolambda–shift − 75 –extsize 150) and obtained 34,460 (E12.5), 31,099 (E13.5), and 69,896 (E15.5) peaks for each sample, respectively. We next built a cell-by-peak count matrix using the fragments and peaks as input based on the function FeatureMatrix from the Signac [56] package. We only retained the spots that were located on the tissue.

We ran each method on the cell-by-peak matrix of spatial ATAC-seq data from mouse embryos to detect spatially variable peaks. For those methods that require normalized data as input, we used TF-IDF (Term Frequency—Inverse Document Frequency) for normalization. Of note, *BOOST-GP* and *GPcounts* failed to produce results after 120 h, and we could not obtain results from *SPARK*due to memory issues. Therefore, these three methods were excluded from this evaluation. We selected the top 20,000 peaks as the spatially variable peaks for each method and used these peaks to cluster the spots. We also used all peaks to obtain a baseline for comparison. Because the true clusters were unavailable, we evaluated the clustering performance by following ref[16]. the spatial chaos score (CHAOS) as follows:

$$CHAOS = \frac{{\sum }_{k=1}^{K}{\sum }_{i,j}^{{n}_{k}}{d}_{kij}}{N}$$

where ${d}_{kij}$ is the Euclidean distance between the spots $i$ and $j$ in the cluster $k$ and $N$ is the total number of spots. A lower CHAOS indicates better spatial continuity.

## Supplementary Information

Additional file 1: Supplementary figures: Figure S1 Visualization of simulated datasets. Figure S2 Comparison of the methods for classification accuracy. Figure S3 Evaluation of statistical calibration of the methods. Figure S4 Visualization of ground truth for spatial domain detection task for DLPFC, OSCC and HER2 datasets. Figure S5 Visualization of clustering results for spatial ATAC-seq data

Additional file 2: Supplementary tables: Table S1 Kendall correlation of each method across all simulated datasets. Table S2: auPRC of each method across all simulated datasets. Table S3: Memory and running time for each method across different numbers of spots. Table S4: Evaluation results of spatial domain detection task. Table S5: Evaluation results of spatial ATAC-seq

## Data Availability

All the datasets used in this study are publicly available. The human DLPFC data were downloaded from http://research.libd.org/spatialLIBD [57]. The HPV-negative oral squamous cell carcinoma (OSCC) data were downloaded from the Gene Expression Omnibus (GEO) with the accession number GSE20825358 [58]. The annotations of OSCC samples were obtained from http://www.pboselab.ca/spatial_OSCC. The HER2 data were obtained from https://github.com/almaan/her2st [59]. Spatial-ATAC-seq data were obtained from GEO with the accession number GSE21499160 [60]. The code for simulating spatial-omics data and running the benchmarked methods is available on GitHub: https://github.com/pinellolab/SVG_Benchmarking [61]. The code for running the pipeline on the Open Problems in Single-Cell Analysis platform is available on GitHub: https://github.com/openproblems-bio/task_spatially_variable_genes [62]. The code has also been deposited in Zenodo: https://zenodo.org/records/16700772 [63].
