# Supplementary material for: Systematic benchmarking of computational methods to identify spatially variable genes
Source: Genome Biol. 2025 Sep 18;26:285. doi: 10.1186/s13059-025-03731-2 (PMC12445034; doi:10.1186/s13059-025-03731-2)
Supplement: Supplementary file 1 — Additional file 1: Supplementary figures: Figure S1 Visualization of simulated datasets. Figure S2 Comparison of the methods for classification accuracy. Figure S3 Evaluation of statistical calibration of the methods. Figure S4 Visualization of ground truth for spatial domain detection task for DLPFC, OSCC and HER2 datasets. Figure S5 Visualization of clustering results for spatial ATAC-seq data [file 13059_2025_3731_MOESM1_ESM.docx]

**Systematic benchmarking of computational methods to identify spatially variable genes**

Zhijian Li^1,2,3#^, Zain M.Patel^1,2,3#^, Dongyuan Song^4^, Sai Nirmayi Yasa^5^, Robrecht Cannoodt^5,6,7^, Guanao Yan^8^, Jingyi Jessica Li^8^, and Luca Pinello^1,2,3^*

^1^Broad Institute of MIT and Harvard, Cambridge, MA, USA.

^2^Molecular Pathology Unit, Center for Cancer Research, Massachusetts General Hospital, Boston, MA, USA.

^3^Department of Pathology, Harvard Medical School, Boston, MA, USA.

^4^Interdepartmental Program of Bioinformatics, University of California, Los Angeles, CA, USA

^5^Data Intuitive, BE.

^6^Data Mining and Modelling for Biomedicine group, VIB Center for Inflammation Research, BE.

^7^Department of Applied Mathematics, Computer Science, and Statistics, Ghent University, BE.

^8^Department of Statistics and Data Science, University of California, Los Angeles, CA, USA

^#^Authors contributed equally

*Corresponding author: Luca Pinello (lpinello@mgh.harvard.edu)

**Supplementary Figures**


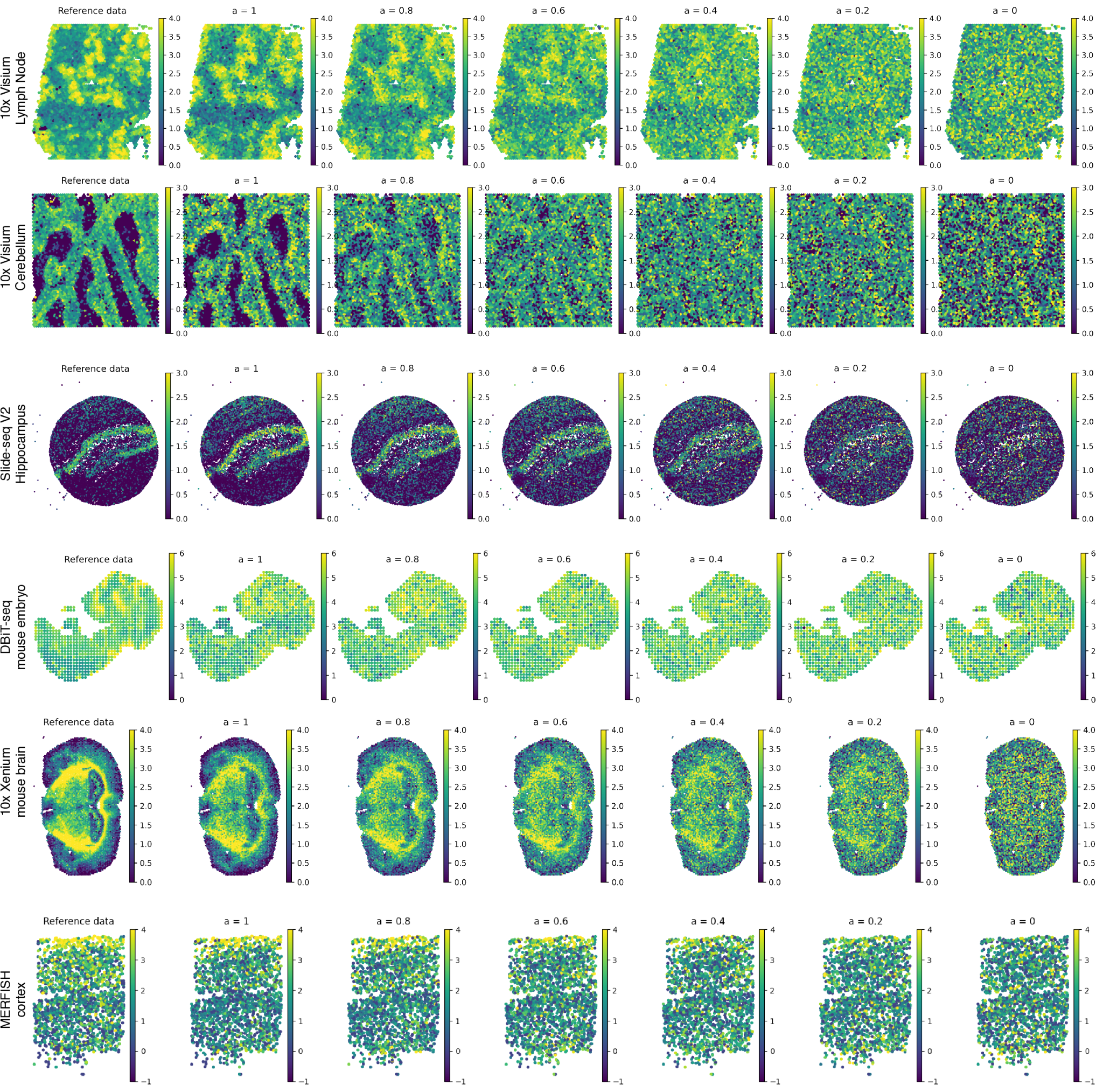


**Fig. S1 Visualization of simulated datasets.** The first column represents real data from different spatial technologies and the rest of the columns represent simulated gene expression with various spatial variability, denoted by $\alpha$. Colors refer to normalized expression profiles.


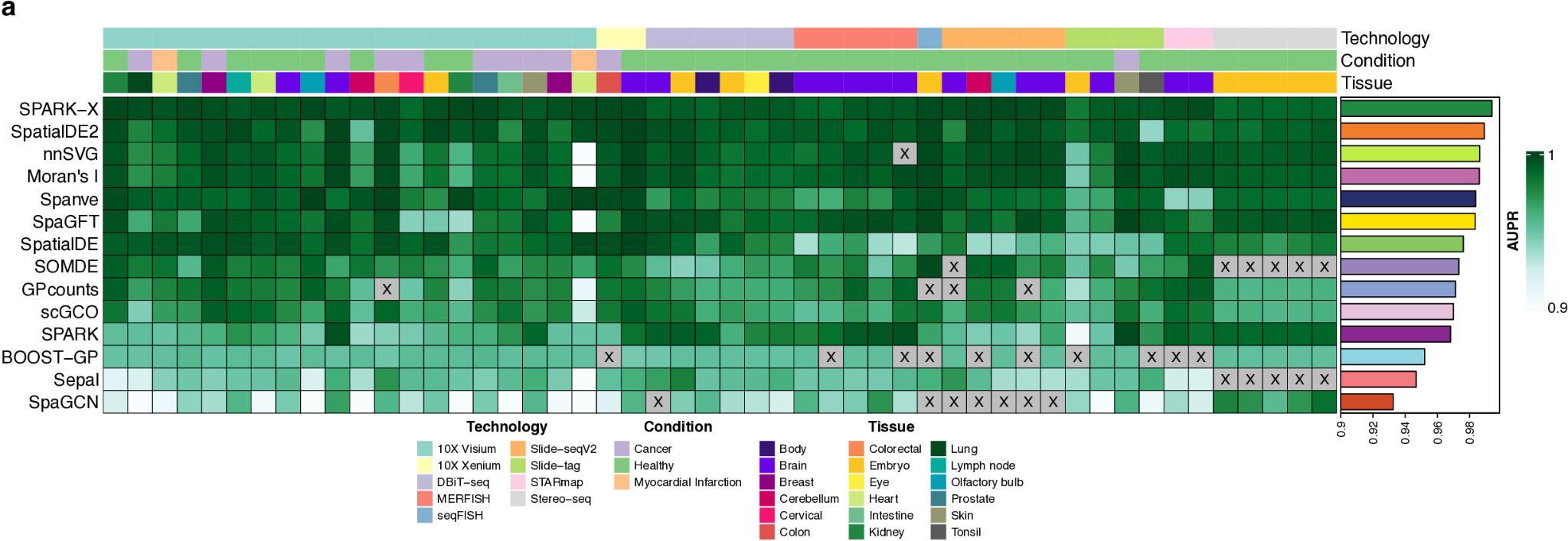


**Fig. S2 Comparison of the methods. a,** Heatmap showing the classification accuracy of each method across all simulated datasets measured by the auPRC. Each row corresponds to a computational method, and each column represents a simulation dataset. A gray color indicates that the method did not produce an output for that dataset. The methods are ordered by their average correlation across all datasets, as shown by the bar plot on the right. The annotations at the top of the heatmap provide details on the spatial technology, tissue type, and biological conditions associated with each dataset.


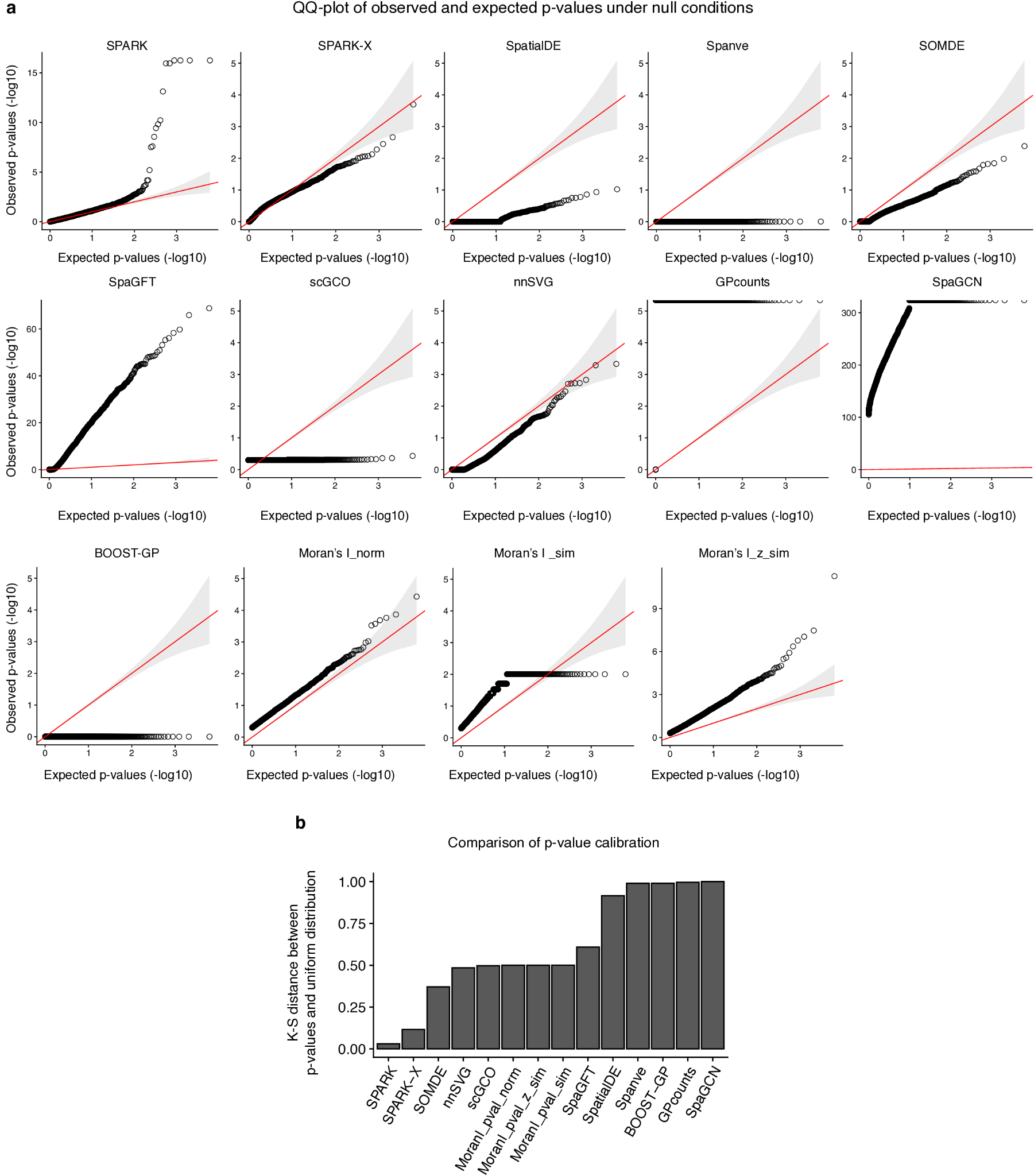


**Fig. S3 Evaluation of statistical calibration of the methods. a,** Quantile-quantile plot of the observed p-values against the expected p-values under the null condition from different methods. **b,** Barplot comparing the K-S distance between observed p-values under null condition and uniform distribution. A lower value represents a better calibrated model.


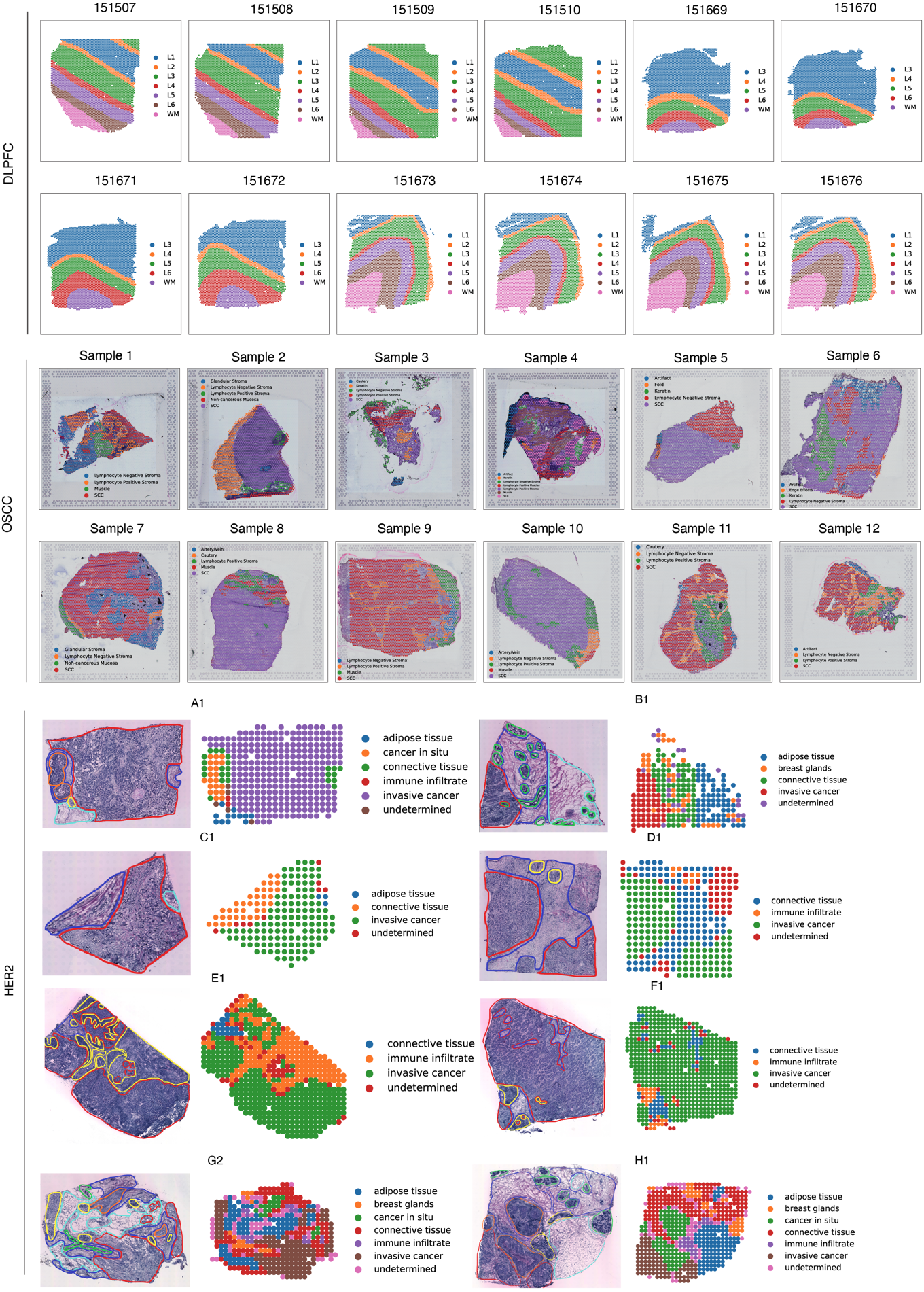


**Fig. S4 Visualization of ground truth for spatial domain detection task for DLPFC, OSCC and HER2 datasets.**

**
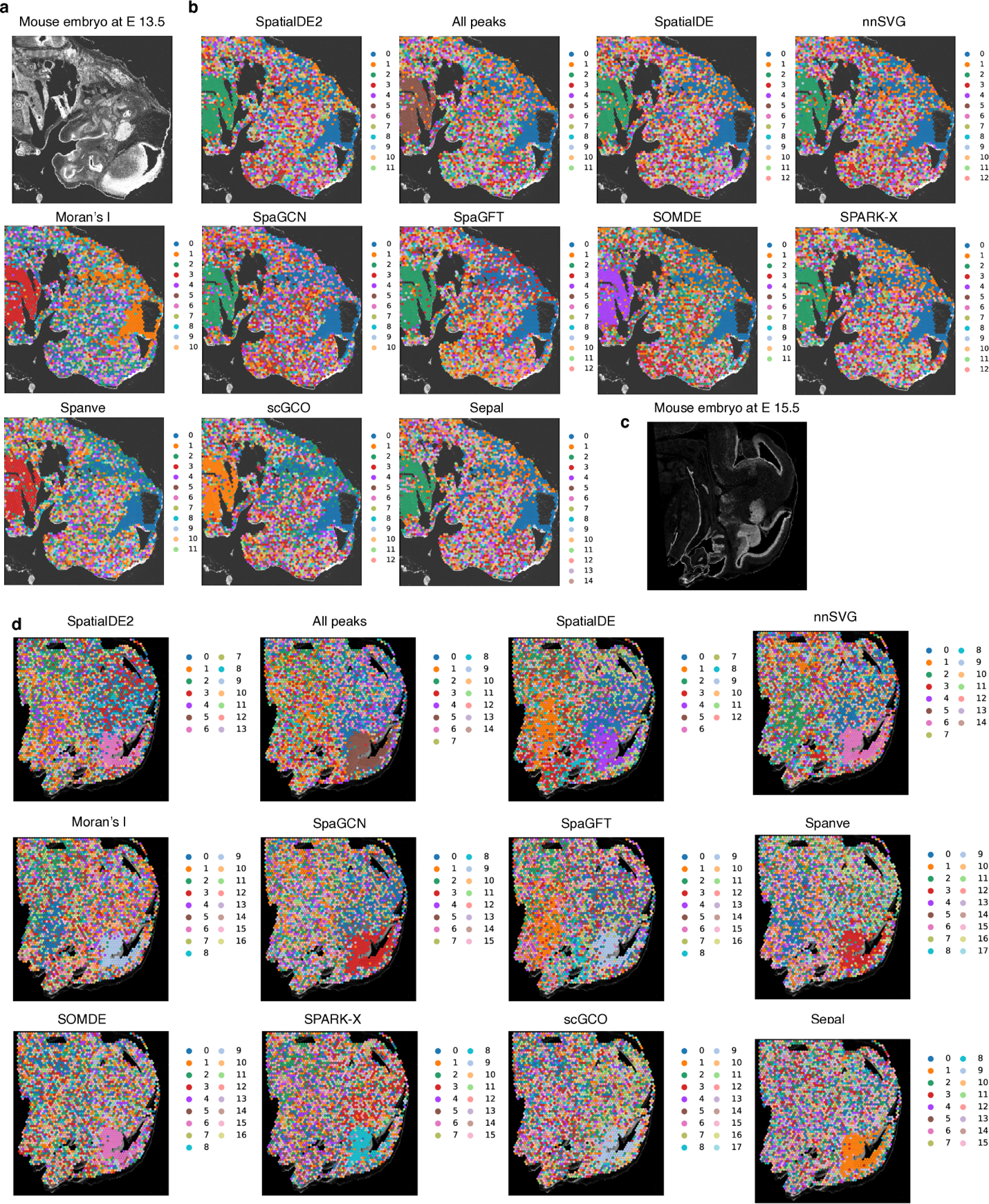
**

**Fig. S5 Visualization of clustering results for spatial ATAC-seq data. a,** Image of a mouse embryo at days of E13.5. **b,** Visualization of obtained clusters by using spatially variable peaks identified by different methods. **c,** Image of a mouse embryo at days of E15.5. **d,** Visualization of obtained clusters by using spatially variable peaks identified by different methods.
